# Clinical Added Value of SARS-CoV-2 Antigen Detection in Blood Samples

**DOI:** 10.3390/diagnostics12102427

**Published:** 2022-10-07

**Authors:** Saoussen Oueslati, Melek Manai Bouokazi, Ikrame Ramdhani, Lélia Escaut, Tài Pham, Souad Ouzani, Nadia Anguel, Sophie Bulifon, Christelle Vauloup-Fellous, Audrey Coilly, Laurence Legros, Magali Guichardon, Nicolas Fortineau, Laurent Dortet, Anne-Marie Roque-Afonso, Thierry Naas

**Affiliations:** 1 Bacteriology-Hygiene Unit, Hôpital Bicêtre, APHP Paris-Saclay, Team ReSIST, INSERM U1184, Université Paris-Saclay, LabEx LERMIT, 94270 Le Kremlin-Bicêtre, France; 2 Service de Virologie, Hôpital Paul-Brousse, APHP Paris-Saclay, and UMR 1193 Physiopathogénèse et Traitement des Maladies du Foie, 94800 Villejuif, France; 3 Unité de Recherche Clinique, Hôpital Bicêtre, 94270 Le Kremlin-Bicêtre, France; 4 Service de Maladie Infectieuse et Tropicale, 94270 Le Kremlin-Bicêtre, France; 5 Service de Médecine Intensive et Réanimation, Hôpital Bicêtre, APHP Paris-Saclay, 94270 Le Kremlin-Bicêtre, France; 6 Service de Pneumologie et Soins Intensifs Respiratoires, Hôpital Bicêtre, APHP Paris-Saclay, 94270 Le Kremlin-Bicêtre, France; 7 Centre Hépatobiliaire, Hôpital Paul-Brousse, APHP Paris-Saclay, 94800 Villejuif, France; 8 Hématologie Clinique, Hôpital Paul-Brousse, APHP Paris-Saclay, 94800 Villejuif, France; 9 Gériatrie, Hôpital Paul-Brousse, APHP Paris-Saclay, 94800 Villejuif, France

**Keywords:** COVID-19, SARS-CoV-2, immunochromatographic assay, antigen, rapid detection, serum

## Abstract

This study evaluated the performances of immunoassays (LFIA and ELISA) designed for SARS-CoV-2 Antigen (Ag)-detection in nasopharyngeal (NP) and serum samples in comparison to RT-PCR. NP samples from patients with respiratory symptoms (183 RT-PCR-positive and 74 RT-PCR-negative samples) were collected from March to April and November to December 2020. Seroconversion and antigen dynamics were assessed by symptom onset and day of RT-PCR diagnosis. Serum samples from 87 COVID-19 patients were used to investigate the added value of Ag quantification, at diagnosis and during follow-up. The sensitivity of COVID-VIRO-LFIA on samples with Ct ≤ 33, considered as the contagious threshold, was 86% on NPs (CI 95%: 79–90.5) and 76% on serum samples (CI 95%: 59.4–88), with a specificity of 100%. Serum N-Ag was detected during active infection as early as day two from symptom onset, with a diagnostic sensitivity of 81.5%. Within one week of symptom onset, diagnostic sensitivity and specificity reached 90.9% (95% CI, 85.1%–94.6%) and 98.3% (95% CI, 91.1%–99.9%), respectively. Serum N-Ag concentration closely correlated with disease severity. Longitudinal analysis revealed the simultaneous increase of antibodies and decrease of N-Ag. Sensitivities of COVID-VIRO-LFIA and COV-QUANTO-ELISA tests on NP and serum samples were close to 80%. They are suitable COVID-19-laboratory diagnostic tests, particularly when blood samples are available, thus reducing the requirement for NP sampling, and subsequent PCR analysis. ELISA titers may help to identify patients at risk of poor outcomes.

## 1. Introduction

Severe acute respiratory syndrome coronavirus 2 (SARS-CoV-2), responsible for the coronavirus disease 2019 (COVID-19), was initially reported in December 2019 in the city of Wuhan, China [1], and has since rapidly spread worldwide, becoming a global health concern [2]. To stem the spread of this pandemic virus, an early and quick diagnosis is crucial to rapidly implement infection control measures [3]. Most of the diagnostic tools used for SARS-CoV-2 detection are based on detection of viral RNA using RT-PCR, defined as the gold standard [4]. However, performing RT-PCR requires special equipment and trained laboratory staff, familiar with molecular techniques. Moreover, molecular tests are costly, time consuming in the absence of automation, and a long time-to-result was reported in the early months of the pandemic [4,5]. Thus, several rapid serological and antigenic diagnostic tests (RDTs), based on lateral flow immunoassay (LFIA) technology, have been developed to detect anti-SARS-CoV-2 antibodies from capillary blood or serum, or SARS-CoV-2 antigens from nasopharyngeal and nasal secretions and saliva. They provide quick results, which are available in 15 minutes, and can be used for point-of care testing (POCT) [5,6]. This unprecedented sanitary crisis has led to a blooming of tests, with hundreds of antibody and antigen detection assays reported on the FIND website [7] either being in use or at some stage of development. The analytical performance and clinical usefulness of several of these assays has not been evaluated, or only on small numbers of samples [4]. Some COVID-19 antigen detection tests have been thoroughly tested and results were disappointing, with a lack of sensitivity in patients with low viral load in nasopharyngeal specimens (i.e., high Ct values of RT-PCR assay) [8,9,10]. However, later generations of LFIA assays, such as the COVID-VIRO^®^, revealed excellent sensitivity of 96.7% (CI, 93.5%−99.9%) as compared to RT-PCR [11] on NP samples. LFIA tests have played an important role in mitigating the effects of the global pandemic with SARS-CoV-2, due to their ability to rapidly detect infected individuals and stop further spread of the virus [5].

RT-PCR analyses for SARS-CoV-2 RNA and immunoassays for SARS-CoV-2 antigen rely mostly on swabs collected from the upper respiratory tract: nasopharyngeal (NP) or nasal swabs, sputum, saliva, or bronchoalveolar lavage, which are heterogeneously composed, more or less well performed, and somewhat ill-defined test material [12]. Immunoassays for SARS-CoV-2 antibodies detection rely on blood samples, which are by far the most used biological material in laboratory diagnostic procedures, and consistencies and variations of this sample material are very well characterized. Viral RNA in the blood is detectable in less than 20% of COVID-19 cases [13]. During the severe acute respiratory syndrome (SARS) epidemic in 2002 to 2004, it was suggested that the nucleocapsid protein (N protein) of SARS coronavirus-1 (SARS-CoV-1) could be detected by enzyme-linked immunosorbent assay (ELISA) in serum samples collected from 95% of infected patients three days after symptom onset [14]. The effectiveness of serum as a specimen for the detection of SARS-CoV-2 antigens has also been evaluated. Li et al. analyzed 50 cases of SARS-CoV-2 nucleic acid-positive and SARS-CoV-2 antibody-negative patients, observing an N protein positivity rate of 76%, suggesting that the serum measurement of SARS-CoV-2 N protein by ELISA can have high diagnostic value for infected patients before the antibody appears, thus shortening the window of serological diagnosis [15]. Other studies have demonstrated N-Ag in blood (serum and plasma) as an interesting biomarker for SARS-CoV-2 infection [16,17,18], with diagnostic sensitivity and specificity of ELISA reaching 81.4% and 99.8%, respectively [19].

In this study, have investigated the clinical added value of Ag quantification by ELISA, at diagnosis and during follow-up. In addition, we have evaluated the biological performances of a lateral flow immunoassay (LFIA) designed for SARS-CoV-2 nucleocapsid (N) antigen detection from nasopharyngeal swabs, on serum samples in comparison to ELISA immunoassay.

## 2. Materials and Methods

### 2.1. Clinical Samples

Nasopharyngeal (NP) samples (eSwabs™-Virocult, Copan, Italy), and serum samples were collected as part of the work-up of respiratory symptoms of patients from two University hospitals located in the southern suburbs of Paris (Bicêtre and Paul Brousse Hospitals). After completion of ordered analysis, NP and serum samples were stored at −80°C and −20°C or +4°C, respectively, until use for research.

NP samples comprised of 183 RT-PCR-positive and 74 RT-PCR-negative samples collected from March to April and from November to December 2020, from 257 patients with respiratory symptoms. A total of 28 additional NP samples from patients infected with known SARS-CoV-2 variants were also tested.

Serum samples from 56 out of the 183 RT-PCR-positive patients were used for rapid antigen testing evaluation in reference to Ag quantification with ELISA. These 64 sera were collected on the same day as the NP sample. In addition, for 18 of these 64 patients, 54 sera were collected at the physician’s discretion during follow-up. For specificity assessment, we used 42 sera collected from May to September 2020 in 42 COVID-19-negative patients, with a negative SARS-CoV-2 RT-PCR in the NP sample, and absence of antibodies to SARS-CoV-2 N antigen. A total set of 183 serum samples from 87 COVID-19 patients (64 with serum collected on the same day as the NP sample, and 19 with serum collected >4 days after diagnosis) were used to investigate added value of Ag quantification, at diagnosis and during follow-up.

### 2.2. N Antigen Detection by Lateral Flow Immunoassays

The LFIA COVID-VIRO (AAZ, Boulogne-Billancourt, France), designed for the detection of SARS-CoV-2 N antigen in NP samples, was used by adding 50 µL of NP samples to 50 µL of buffer provided in the kit. Then, the whole mix was loaded onto the cassette. The COVID-VIRO assay was also used to detect N antigen from serum samples, with minimal modification: 100µL of serum were used on the LFIA instead 50 µL of NP sample.

LFIA assays were conducted retrospectively with personnel blinded to all other test results and according to manufacturer instructions. NP samples were removed from −80°C storage ≤1 h and placed at room temperature. No significant change in the test performances was observed using NP samples before and after, without freezing and thawing (data not shown).

### 2.3. SARS-CoV-2 Antibody Detection by Lateral Flow Immunoassays

LFIA COVID-PRESTO (AAZ) was used, according to manufacturer instructions, to detect the serological IgG and IgM response against N and S proteins.

### 2.4. SARS-CoV-2 Detection from NP Samples by RT-PCR

SARS-CoV-2 RT-PCR results were obtained using two different assays targeting ORF1 and E genes or RNA-dependent RNA polymerase (RdRp) and N genes of SARS-CoV-2 (6800 SARS-CoV-2 test, Roche Molecular Systems, Branchburg, NJ, USA, and Alinity m SARS-CoV-2 assay, Abbott Molecular, Des Plaines, IL, USA).

### 2.5. Quantification of N Antigen Levels from Serum Samples

N-antigenemia levels were determined from serum samples with a CE-IVD ELISA microplate assay, the COV-QUANTO kit (AAZ, Boulogne-Billancourt, France), following manufacturer instructions. This assay has been evaluated elsewhere [11,16]. The cut-off limit was presented at 2.98 pg/mL, according to manufacturer recommendations.

### 2.6. Data Analysis

The sensitivity and specificity values of evaluated assays were calculated with their respective confidence intervals (95% CI) using a free software VassarStats [20].

### 2.7. Ethics

Reclassifications of biological remnants into research material after completion of the ordered virological tests were obtained under number DC 2009-965 and received ethical approval of the CPP Ile de France 7 (N°CO-15-000) and N° IDRCB: 2020-A01442-37, in accordance with French law. The planning, conduct, and reporting of studies was in line with the Declaration of Helsinki as a retrospective non-interventional study with no addition to standard care procedures.

## 3. Results

### 3.1. LFIA Assays for SARS-CoV-2 Antigen Detection in Nasopharyngeal Specimens

The COVID-VIRO assay was negative in 73/74 RT-PCR-negative samples. The overall specificity was 99% (CI 95%: 92−99). The 183 NP samples from COVID-19 patients were classified in categories according to NP viral load, reflected by RT-PCR Ct results. The overall sensitivity of COVID-VIRO in reference to RT-PCR was 75% (CI 95%: 68.4−81.3). However, the COVID-VIRO test was able to detect high (Ct < 25), medium (25 ≤ Ct < 33), and low (33 ≤ Ct) viral loads with 99% (*n* =73; CI 95%: 91.7−99.9), 75% (*n* = 85; CI 95%: 64.5−83.7), and 8% (*n* = 25; CI 95%: 1.39−27.5) sensitivity, respectively (Table 1). In addition, it’s sensitivity in detecting potentially infectious samples (Ct ≤ 33) was 86% (*n* = 160; 79−90.5) [21]. Similar results were recently observed with two other LFIA assays for SARS-CoV-2 antigen detection [4,22].

### 3.2. LFIA Assays for SARS-CoV-2 Antigen Detection with Emerging Variants

Currently, several lineages of SARS-CoV-2 are emerging and circulating globally [23,24]. The most common variants are VOC 202012/01 (known as 20I/501Y.V1 or B.1.1.7 identified in the United Kingdom) [24], the variant 501Y.V2 (identified in South Africa [25]), and the P.1 variant (identified in Brazil [26]). These variants are considered a threat because of their high transmissibility due to mutations of the spike protein. Furthermore, variants with E484K are considered to be potentially able to escape the immune response [27]. In this study, we evaluated the detection, with the LFIA assay, of some of SARS-CoV-2 variants: VOC 202012/01 (*n* = 10), 501.V2 (*n* = 15), L452R; N501Y (*n* = 1), and E484K (*n* = 2) variant. As expected, and taking into account Ct values, all these variants were efficiently detected (data not shown).

### 3.3. SARS-CoV-2 Antigen Detection in Serum Samples by LFIA and ELISA Assays

N antigenemia by ELISA and by the COVID-VIRO assay was undetectable in all 42 sera from COVID-19-negative patients. The overall specificity of COVID-VIRO was 100 % (CI 95%: 92−100). The sensitivity of COVID-VIRO antigen detection in serum was evaluated on 56 samples of COVID-19 patients, collected on the same day as the NP sample used for diagnosis. The overall sensitivity of antigen detection by ELISA and LFIA in reference to RT-PCR was, respectively, 66% (CI 95%: 52−77.8) and 59% (CI 95%: 45−71.6), while sensitivity in detecting potentially infectious patients (Ct ≤ 33, [18]) was 87% (CI 95%:71.1−95.1) and 76% (CI 95%: 59−88), respectively (Table 1). Thus, the sensitivity and specificity of the two assay formats for antigen detection appeared similar.

**Figure 1:**
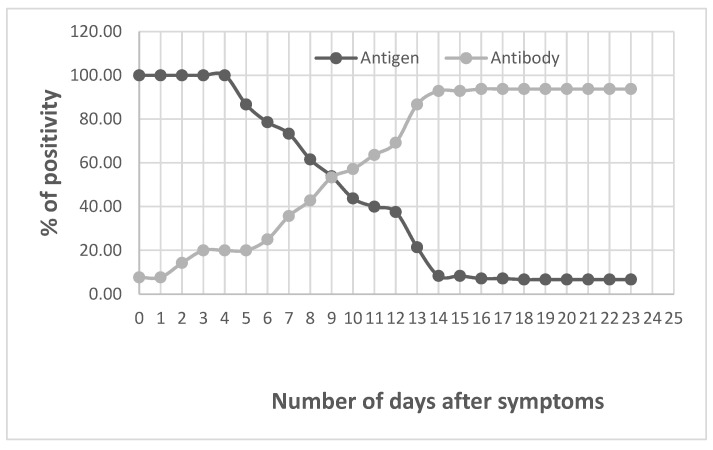
Serology and N-antigen detection in serum of patients after symptoms onset.

For 18 of these patients, serially taken serum samples (over one-month period) were available to observe both the persistence of antigens in the serum and match it with the seroconversion by using the LFIA COVID-PRESTO (Figure 1). All results obtained by LFIA were confirmed by ELISA (data not shown). Thus, we noted that until the fifth day after symptoms onset, all the patients had antigens in the serum. Then, the number of positivity decreased until day 13 when the seroconversion turned maximum.

### 3.4. Added Value of SARS-CoV-2 Antigen Detection in Serum Samples

**Figure 2:**
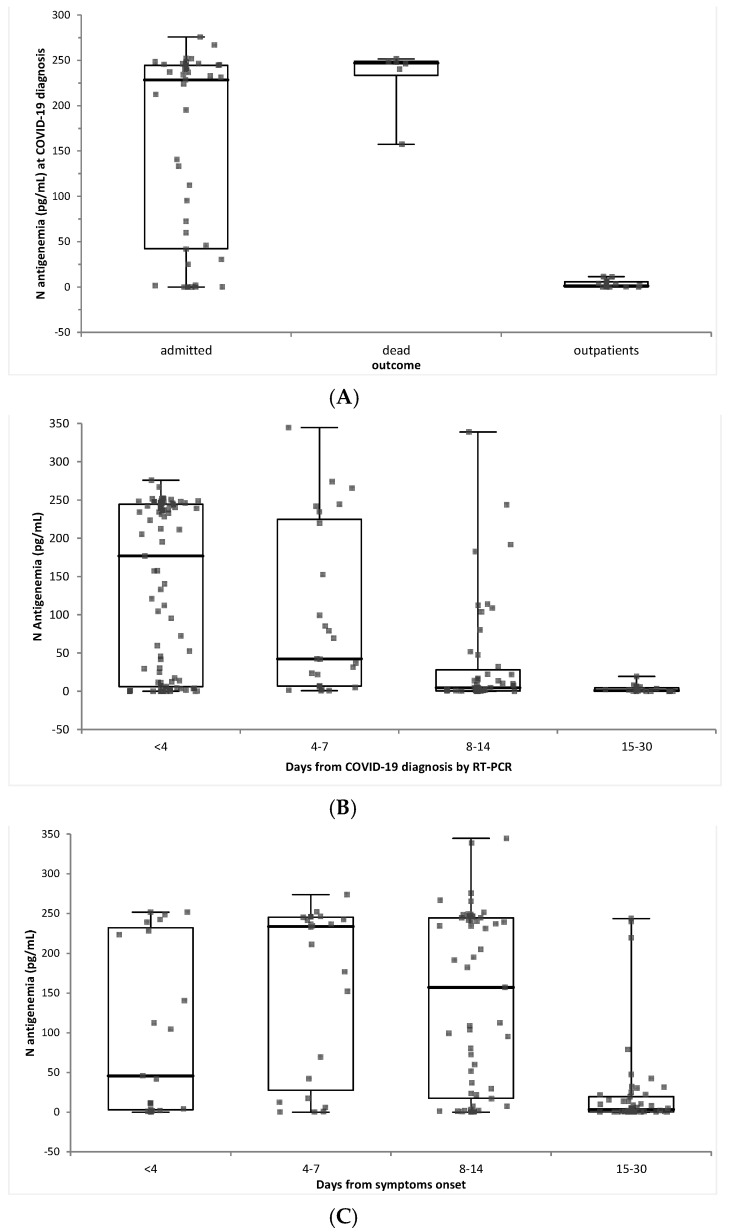
N antigenemia (pg/mL) according to (**A**). patients’ outcome, (**B**). days after COVID-19 diagnosis, and (**C**). days after symptoms onset.

We have shown that Ag detection, either by LFIA or by ELISA, had sensitivities of 76% and 87%, compared to RT-PCR testing at diagnosis used as reference. A set of 64 COVID-19 patients, with available sera collected 0−3 days after COVID-19 diagnosis by NP RT-PCR, were evaluated using the quantitative ELISA immunoassay COV-QUANTO: 15 were outpatients, with a favorable outcome, 43 were admitted either in ICU (*n* = 9) or in conventional hospitalization wards (*n* = 36), and were then discharged, and 6 died during hospitalization. N antigenemia was significantly different according to these outcomes: it was lower in outpatients (3.16 +/− 4.1 pg/mL) than in admitted patients (157.4 +/− 104.4 pg/mL) or in patients who died (231.9 +/− 36.7 pg/mL) (*p* < 0.001) (Figure 2A).

Follow-up samples were available for these 64 patients and additional patients sampled >4 days after diagnosis were available to assess the kinetics of N antigenemia (183 samples from 87 patients). Serum antigenemia was maximal within 4 days of COVID-19 diagnosis by RT-PCR, and peaked 4−7 days after symptoms onset (Figure 2B,C).

## 4. Discussion

The COVID-VIRO test is a rapid SARS-CoV-2 antigen assay for the first-line diagnosis of COVID-19 from NP samples, as a screening in symptomatic patients. This assay is complementary to the currently used molecular techniques. Rapid results of NP antigen with COVID-VIRO test should easily detect contagious people with high virus level, while the sensitive PCR tests additionally detect low level of virus, or nucleic acids, in people who are no longer or less contagious. RT-PCR is a valuable tool and remains the gold standard for SARS-CoV-2 detection, even though clinical performances are debated relating to quality of the sampling method. In a recent systematic review, false-negative PCR rates of between 2% and 33% were described [28], but this is also true for antigen testing from NP samples. On the other hand, rates of operational false-positive swab tests in the UK during the first epidemic wave were estimated to be between 0.8% and 4.0% [29,30], due to the very large number of samples tested and the high rate of positivity, which can be a source of contamination. This rate could translate into a significant proportion of false-positive results daily. Indeed, positive PCR results with high Ct values may reflect residual RNA after days or weeks of infection, or inter-sample contamination from a highly positive patient tested in the same PCR series [30,31].

LFIA antigen testing is rapid, robust, cost-efficient, and helps to increase the number of tests performed, even though they have lower sensitivity compared to PCR. In addition, this test could be helpful in countries or areas where PCR testing is still scarce. The main limitation of antigenic testing is that sensitivity directly depends on the delay between symptoms onset and the sampling, with high sensitivity for the first days of infection when RT-PCR is also more accurate in predicting infectivity [25]. Indeed, the sensitivity value of COVID-VIRO on NP samples with Cts ≤33 was 86% (CI 95%: 79−90.5%). These results are comparable to those of Hingrat et al. that showed that with high nasopharyngeal viral loads, i.e., Ct values below 30 and 33, only 1/50 and 4/67 tested negative for N-antigenaemia, respectively [16]. In addition, they have shown this with hospitalized patients with symptoms compatible with SARS-CoV-2 infection, but with a negative nasopharyngeal RT-PCR, 8/12 presented positive N-antigenaemia. These results were confirmed by exploration of the lower respiratory tract for six of these eight patients, that revealed positive RT-PCR in five cases. These differences between N-antigenaemia and RT-PCR results might be linked to the complex biological matrixes, NP or lower respiratory samples, that contain inhibitors of RT-PCR reactions. Sensitivities of RT-PCR and immunological assays with NP samples have been related to the quality of the sampling method, indeed, as sampling may generate discomfort and pain, the sampling may be done only in posterior nose cavity [28]. On the other hand, blood sampling is a standardized procedure, offering technical and practical advantages, and together with the diagnostic value of serum N-Ag, it may meet unsatisfied diagnostic and prognostic needs during the pandemic.

This study showed that the highest values of N antigenemia were observed at COVID-19 diagnosis, and that antigenemia peaked 4−7 days after symptoms onset. Sensitivities of LFIA and ELISA antigens on serum are close to 80% and may help to identify patients at risk of poor outcomes. Our work further supports serum N-Ag as a biomarker for SARSCoV-2 acute infection, with high diagnostic sensitivity and specificity for ELISA and LFIA compared to viral RNA in NP samples. There is a correlation between serum N-Ag concentrations and disease severity and an inverse relationship of N-Ag and Abs. For instance, this could be the case for infected patients before the antibody appears, thus shortening the window for serological diagnosis. Another main advantage of N antigenemia resides in the fact that these tests might be used as point-of-care testing (POCT) devices on-site of sampling or directly by the patients themselves. However, further evaluation directly focusing on finger blood picking is required to turn these assays into simple POCT assays.

## Figures and Tables

**Table 1:** Sensitivity (%) of the COVID-VIRO LFIA assay (AAZ) in detecting N antigen from nasopharyngeal and respective serum samples, classified by subgroups of NP viral load: high (Ct < 25), medium (25 ≤ Ct < 33), and low (33 ≤ Ct). Sensitivity of the COV-QUANTO assay is also shown. CI 95% are indicated in brackets. Ct values correspond to those of either the PCR Roche 6800 and/or Abbott Alinitym.

	High Viral Load (Ct < 25)	Medium Viral Load (25 ≤ Ct < 33)	Low Viral Load (33 ≤ Ct)	Global Results 11 ≤ Ct ≤ 41, (MED Ct 26.8)	Results Considering Contagious Threshold (Ct ≤ 33) ^a^
LFIA on nasopharyngeal samples	99% *n* =73 (91.7–99.9)	75% *n* = 85 (64.5–83.7)	8% *n* = 25 (1.39−27.5)	75% *n* = 183 (68.4–81.3)	86% *n* = 160 (79−90.5)
LFIA on serum samples	67% *n* = 18 (41.1−85.6)	85% *n* = 20 (61.1−96)	22% *n* = 18 (7.37−48.1)	59% *n* = 56 (45−71.6)	76% *n* = 38 (59.4−88)
ELISA N-Ag detection in Serum samples	83% *n* = 18 (57.7–95.6)	90% *n* = 20 (66.9–98.2)	22% *n* = 18 (7.4–48.1)	66% *n* = 56 (52−77.8)	87% *n* = 38 (71.1–95.1)

^a^ Commonly accepted threshold for infectiousness of COVID-19 patient [21].

## Data Availability

Data supporting reported results may be available upon request.
